# Parenthood and psychological distress among English Millennials during the second wave of the COVID-19 pandemic: evidence from the Next Steps cohort study

**DOI:** 10.1007/s00127-022-02392-x

**Published:** 2022-11-27

**Authors:** B. Chen, A. McMunn, T. Gagné

**Affiliations:** 1 Department of Epidemiology and Public Health, University College London, Gower Street, London, WC1E 6BT UK; 2 International Centre for Lifecourse Studies in Society and Health, London, UK

**Keywords:** England, Parenthood, Mental health, Young adults, General health questionnaire, Next Steps cohort

## Abstract

**Purpose:**

The COVID-19 pandemic led to disproportionate mental health responses in younger adults and parents. The aim of the study was to investigate how Millennial parents’ experiences were associated with psychological distress over the first year of the pandemic.

**Methods:**

We examined data in September 2020 (*n* men = 994; *n* women = 1824) and February 2021 (*n* men = 1054; *n* women = 1845) from the Next Steps cohort study (started ages 13–14 in 2003–04). In each wave, we examined differences in GHQ-12 scores between parent groups defined by the age and number of children, adjusting for background characteristics at ages 13–14, psychological distress at ages 25–26, and other circumstances during the pandemic. We also examined if differences varied by work status, financial situation before the outbreak and relationship status.

**Results:**

Whereas mothers with one or two children and children aged 0–2 reported less distress than non-mothers in September 2020, there were no such differences in February 2021. Fathers with three or more children reported more distress in February 2021. Compared with non-fathers who worked, fathers were also disproportionally distressed if they were working with one child or with children aged 2 or less in September 2020.

**Conclusion:**

The distribution of psychological distress among Millennial parents and non-parents has varied by age, sex, parenting stage, work status and the timing of the pandemic. Generous family policies are needed, with special attention dedicated to parents combining work and family responsibilities.

**Supplementary Information:**

The online version contains supplementary material available at 10.1007/s00127-022-02392-x.

## Introduction

Parenthood is one of the key life events that individuals may make into their transition to adulthood [1]. Parenthood is a complex process with a range of stressors requiring multiple social and economic resources to cope through, with ethnicity, family background, and education each having meaningful influences on its experience [2–5]. During this life period, even with economic and social support, conditions such as psychological distress, physical inactivity, and low sleep quality are likely to worsen [6, 7]. Taking these into account, some parenthood transitions, such as becoming a parent at an early age and raising children without a partner, may negatively influence social and health trajectories over the life-course [8]. Poor adaptation at this stage not only has adverse effects on parents, but also on the wellbeing of their offspring [9]. In particular, psychological distress is associated with worse parenting practices such as negative daily interactions with children that may subsequently affect their lifelong development [10]. Therefore, it is crucial to make sure that new, young parents are properly supported and may thrive. Two elements, however, complicate our understanding of the distribution of mental health among young parents at the moment.

First, the causal relationship between parenthood and psychological distress is nuanced and varies by sex, age, and parenthood trajectory (i.e. the timing and number of children), leading the evidence on their association to remain inconsistent [5, 11, 12]. Therefore, instead of comparing parents to non-parents, it is important to take individual changes across parental stages over time into account when defining parent groups [4, 13]. Using this approach, younger parents, mothers in particular, who take care of multiple, young children have been highlighted as the key risk group for the development of psychological distress among parents [14–16]. The impact of these parenthood experiences is further determined by conditions across work, housing, and relationships [17]. In particular, higher education and family income have been highlighted as protective factors, and so has being in a relationship, especially when it is stable and happy [18–21].

Second, the experience of parenthood has been dramatically affected by the COVID-19 pandemic, further limiting the generalisability of previous evidence. In response to the public health measures enforced over time, schools and nurseries have been closed, children no longer had access to group activities and playgrounds, and approximately one-third of parents had to adapt their work to take care of their children [22]. For many parents, it has been a difficult task to keep children safe and busy at home [23]. The economic downturn sparked by the pandemic has also led many parents to experience decreased wages due to reduced work hours, job loss, or continued unemployment, including those living in low-income households [24]. Finally, there has also been evidence of increased gender inequalities in the division of childcare, with mothers having to spend more time on housework and childcare than fathers during the lockdown [22, 25]. This increased workload has been associated at the start of the pandemic with higher levels of psychological distress and financial insecurity in mothers and fathers, and disproportionally affected mothers who were not partnered and worked at the same time [22, 26].

### Objectives

Younger parents and parents of younger children are likely to have been disproportionally affected by the immediate social and economic consequences of the COVID-19 pandemic [27–31]. Compared to other age groups, younger adults have been more likely to experience distress, lose their job, and turn to more precarious work during the pandemic [32]. Few studies, however, have focussed on understanding the magnitude of the burden of mental health in young parents since the start of the pandemic. Past evidence on parenthood and mental health likely only partially applies to experiences during the pandemic. In addition, a significant portion of studies that examined the distribution of mental health in 2020–21 used data collected around the first COVID-19 wave (i.e. April–May 2020), precluding us from understanding whether inequalities in response to the initial shock have persisted over time.

To shed light on how the experiences of young parents differed over the course of the pandemic, this study examines differences in psychological distress across non-parents and parent groups using data from Next Steps, a longstanding cohort study that followed a large sample of English Millennials in 2020–21. We leverage data on mental health, family, and finances collected in adolescence (ages 13–14) and young adulthood (ages 25–26), and at two points in September–October 2020 and February–March 2021 when participants were aged 30–31. Specifically, this study examines: (1) differences by parent status defined by the age and number of children; (2) whether differences further varied by work status, financial conditions before the pandemic, and relationship status (i.e. effect modification). In keeping with gendered differences in parenthood and pandemic responses, associations were examined in men and women separately.

Based on the assumptions that parenting is gendered, parents report more wellbeing in the years around childbirth, and lockdown restrictions affected parenting in different ways in keeping with children’s age, we expect: (1) higher distress among parents compared with non-parents, particularly in women; (2) higher distress among parents with older children aged > 2; (3) higher distress among parents in February–March 2021 during the end of the second COVID-19 wave compared with September–October 2020; (4) higher distress among parents who were more vulnerable, as defined by financial and relationship circumstances.

## Methods

### Data

Next Steps is a nationally representative longitudinal cohort study of 15,770 individuals born in 1989–90. After the baseline survey at ages 13–14 in 2003–04, cohort members were interviewed every year until ages 19–20 in 2010. In 2015–16, cohort members were contacted once again to find out how their lives had turned out at ages 25–26 [33]. In 2020–21, cohort members were invited to take part in a COVID-19 sub-study to gather information on the impacts of the pandemic [34]. Wave 1 took place in May 2020 to capture responses following the first wave peak, Wave 2 took place in September–October 2020, and Wave 3 took place in February–March 2021 [34]. Whereas contact in Wave 1 was only made by email over a short period of time, Waves 2 and 3 also used mail and phone, financial incentives, and a longer fieldwork period to ensure better response rates. This study therefore focusses on Waves 2 and 3. Among eligible cases, 3664 cohort members participated in Wave 2 (RR = 31.8%), and 4239 participated in Wave 3 (RR = 34.3%).

### Measures

Psychological distress was measured at each wave using the 12-item General Health Questionnaire (GHQ), a validated scale for measuring non-specific mental distress that has been widely used in community and non-clinical settings (see items in Supplementary Table 1) [35]. We used the Likert score, recoded by adding items’ responses into a composite score from 0 (least distressed) to 36 (most distressed). Whereas the COVID-19 surveys measured other indicators of mental health, the GHQ was the only variable measured in earlier waves, making this choice a stronger analytic strategy.

Parenthood status was first assessed with an item asking if participants currently lived with children (including adopted children, stepchildren, foster children, adult children, or any other children). The age and number of children was reported in a household grid questionnaire. Given that less than 1% of participants had more than three children, we recoded participants into: (1) no children; (2) one child; (3) two children; (4) three or more children. Similarly, given that less than 10% of parents had children aged over 5, we recoded participants based on the age of the youngest child into: (1) no children; (2) aged 2 or less; (3) aged 3–4; (4) aged 5 or more.

The following covariates were considered based on previous studies and data across Next Steps waves [3, 11, 16, 22, 36–38]: at ages 13–14, (1) ethnicity (White/non-White), (2) home ownership (owner/not owner); at ages 25–26, (3) education (no post-secondary education/post-secondary education below degree/degree), (4) social class based on the National Statistics Socio-economic classification (managerial and professional/intermediate and self-employed/lower supervisory, semi-routine, and routine/not working), (5) psychological distress (GHQ-12 Likert score). Other covariates considered in Waves 2 and 3 were: (6) living arrangements (alone/with other adults), (7) relationship status (in a couple/no), (8) working status (employed or self-employed and working/no), and (9) financial situation before the outbreak based on the item “In the three months before the Coronavirus outbreak, how well would you say you personally were managing financially?” (comfortably/less than comfortably).

### Statistical analyses

We first present the distribution of GHQ-12 scores, parenthood categories, and covariates in men and women in descriptive tables. The distribution of variables is also presented across parent groups for Wave 3 in Supplementary Tables 2.1 and 2.2.

We then report results from linear regression models to examine differences in psychological distress between parent groups, using non-parents as the reference category, in the two waves separately. For each of the two parent variables, two models were fitted: (1) Model 1 included covariates measured at ages 13–14 and 25–26; (2) Model 2 also included covariates measured at the same time in Wave 2 or 3. We also produced adjusted Wald-type tests to test the joint significance of coefficients. We then tested interactions to assess the potential variation in differences between parent groups according to circumstances during the pandemic. Results from the interactions are detailed in the Supplementary Tables 3.1–3.3.

We entered the two parent variables in separate models to limit collinearity concerns. Similarly, we did not include the two “living arrangements” and “relationship status” variables in models among men because over 97% of fathers were partnered and living with at least one other adult across waves. For the same reason, interactions by work status and financial situation before the outbreak were tested in both sexes, but interactions by relationship status were tested in women only.

Analyses were run in complete-case samples in each wave separately, integrating weight, cluster, and stratification variables, using Stata 17 [39]. In Wave 2, this resulted in a sample size of 994 men and 1824 women. In Wave 3, this resulted in a sample size of 1054 men and 1845 women. Given the number of tests, we use a significance level of 0.01 to interpret findings.

### Sensitivity analysis

While the use of cross-sectional models precludes us from distinguishing trajectories of psychological distress, we examined the robustness of cross-sectional models tested in Wave 2 by reproducing them in a longitudinal framework, i.e. regressing GHQ at Wave 3 on parent variables at Wave 2 controlling for GHQ at Wave 2 and other covariates. Results corroborate findings from cross-sectional models, and are presented at the end.

## Results

### Description of samples in Sep–Oct 2020 and Feb–Mar 2021

Table 1 presents the distribution of variables. In September–October 2020, GHQ scores averaged 13.4 in men and 14.4 in women. In February–March 2021, GHQ scores slightly increased to 13.9 in men and 15.4 in women. In comparison, GHQ scores at ages 25–26 in 2015–16 were 11.2–11.3 in men and 12.2–12.3 in women.

**Table 1:** Sample characteristics

	Wave 2 September–October 2020			Wave 3 February–March 2021		
	Total	Men	Women	Total	Men	Women
Variable	*N* = 2818	*N* = 994	*N* = 1824	*N* = 2899	*N* = 1054	*N* = 1845
	% Weighted	% Weighted	% Weighted	% Weighted	% Weighted	% Weighted
GHQ-12 score, mean	13.96	13.43	14.43	14.74	13.91	15.40
Number of children						
No children	60.31	72.55	49.27	61.82	72.11	53.61
1	18.26	12.42	23.54	17.47	14.72	19.66
2	15.17	11.45	18.52	14.99	8.70	20.02
3 +	6.26	3.59	8.67	5.71	4.46	6.71
Age of the youngest child						
No children	60.31	72.55	49.27	61.82	72.11	53.61
0–2 years	25.28	17.78	32.05	19.59	17.21	21.49
3–4 years	6.67	4.67	8.47	8.40	5.23	10.93
5 + years	7.74	5.00	10.21	10.19	5.45	13.97
Ethnicity						
White	87.47	87.25	87.67	69.69	71.88	67.94
Non-White	12.53	12.75	12.33	30.31	28.12	32.06
Homeownership at ages 13–14						
Yes	76.63	77.76	75.62	75.86	79.93	72.61
No	23.37	22.24	24.38	24.14	20.07	27.39
Social class at ages 25–26						
Never worked, unemployed, or other	17.56	16.08	18.89	18.45	16.68	19.87
I: Managerial and professional	40.32	39.12	41.40	41.97	43.18	41.00
II: Intermediate and small employers	17.98	15.74	20.01	18.43	16.71	19.81
III: Technical and (semi-)routine	24.14	29.05	19.70	21.15	23.43	19.33
Educational attainment at ages 25–26						
Secondary education or less	38.97	38.92	39.02	36.29	37.74	35.14
Post-secondary education below degree	18.79	19.02	18.59	18.57	17.98	19.04
Degree or above	42.24	42.06	42.39	45.14	44.28	45.82
GHQ-12 at ages 25–26, mean	11.77	11.28	12.22	11.80	11.16	12.31
Work status in 2020–21						
Working	74.32	78.79	70.30	73.37	83.14	65.58
Not working	25.68	21.21	29.70	26.63	16.86	34.42
Living arrangements in 2020–21						
Living alone	12.74	10.25	14.99	15.95	14.21	17.34
Living with other adults	87.26	89.75	85.01	84.05	85.79	82.66
Relationship status in 2020–21						
No partner	20.88	21.93	19.92	20.40	19.92	20.78
With a partner	79.12	78.07	80.08	79.60	80.08	79.22
Fin. sit. before outbreak in 2020–21						
Living comfortably	37.63	38.49	36.86	38.78	39.64	38.09
Living less than comfortably	62.37	61.51	63.14	61.22	60.36	61.91

Estimates were produced in the wave-specific complete-case samples

Next Steps cohort COVID-19 survey waves 2–3, ages 30–31. England, 2020–2021

Across waves, around 27–28% of men and 46–51% of women were parents, with 45–53% of fathers and 42–46% of mothers having one child, and 62–64% of fathers and 46–63% of mothers having their youngest children aged 2 or less. There was relatively little change in circumstances during the pandemic across the two waves. The majority of participants worked (M: 78–83%; W: 66–70%), lived with at least one other adult (M: 86–90%; W: 80–83%), were partnered (M: 78–80%; W: 80–80%), but also lived less than comfortably before the outbreak (M: 61–62%; W: 62–63%).

The lower proportion of mothers with young children aged 2 or less in Wave 3 compared with Wave 2 may point to differences in non-response across waves that were not fully mitigated by weighting. Supporting this, participants were also more likely to be not White in Wave 3 (30%) compared with Wave 2 (13%). Other background covariates, however, were very consistent across waves.

### Psychological distress between parent groups in Sep–Oct 2020 and Feb–Mar 2021

Tables 2 and 3 report the results from the multivariable models estimating differences in GHQ scores by parent variables in men and women, using non-parents as the reference category. We report results for each wave sequentially.

**Table 2:** Association between parent characteristics and psychological distress (GHQ 0–36 score) in men. Next Steps cohort COVID-19 survey waves 2–3, ages 30–31. England, September 2020 to March 2021

	Wave 2 Sep.–Oct. 2020				Wave 3 Feb.–Mar. 2021			
Variables	Model 1 + partially adjusted		Model 2 + COVID covariates		Model 1 + partially adjusted		Model 2 + COVID covariates	
	*B*	95%CI	*B*	95%CI	*B*	95%CI	*B*	95%CI
Number of children	Joint *p* = 0.177		Joint *p* = 0.192		Joint *p* = 0.359		Joint *p* = 0.200	
(ref. No child)	–	–	–	–	–	–	–	–
1	− 1.31	− 2.78, 0.16	− 1.05	− 2.45, 0.35	0.57	− 1.03, 2.18	0.40	− 1.08, 1.89
2	− 0.42	− 1.95, 1.11	− 0.57	− 2.21, 1.07	0.51	− 1.03, 2.05	0.25	− 1.27, 1.76
3 +	0.69	− 0.98, 2.37	1.04	− 0.72, 2.80	2.28	− 0.42, 4.97	2.64	0.21, 5.07
Age of youngest child	Joint* p* = 0.214		Joint* p* = 0.312		Joint* p* = 0.278		Joint *p* = 0.329	
(ref. No child)	–	–	–	–	–	–	–	–
0–2	− 0.32	− 1.47, 0.82	− 0.27	− 1.53, 0.99	0.59	− 0.82, 2.01	0.65	− 0.69, 1.98
3–4	− 0.17	− 2.18, 1.83	− 0.20	− 1.99, 1.58	0.26	− 1.48, 2.00	− 0.16	− 1.83, 1.50
5 +	− 2.48	− 4.79, − 0.18	− 2.12	− 4.33, 0.09	2.19	− 0.03, 4.41	1.87	− 0.27, 4.00

Model 1: adjusted for ethnicity, housing tenure at ages 13–14, and educational attainment, social class and GHQ score at ages 25–26

Model 2: also adjusted for financial situation before outbreak, and employment status at the current wave

Coefficients are linear regression betas, representing the differences in GHQ score between categories. A higher GHQ score represents a higher level of distress

Joint *p* values are adjusted Wald-type tests for all three coefficients

All analyses were weighted and adjusted for the survey design and non-response

**Table 3:** Association between parent characteristics and psychological distress (GHQ 0–36 score) in women

	Wave 2 Sep.–Oct. 2020				Wave 3 Feb.–Mar. 2021			
Variables	Model 1 + partially-adjusted		Model 2 + COVID covariates		Model 1 + partially-adjusted		Model 2 + COVID covariates	
	*B*	95%CI	*B*	95%CI	*B*	95%CI	*B*	95%CI
Number of children	Joint *p* = 0.102		Joint *p* = 0.024		Joint *p* = 0.254		Joint *p* = 0.553	
(ref. No child)	–	–	–	–	–	–	–	–
1	− 0.85	− 1.81, 0.11	− 1.17	− 2.13, − 0.20	− 0.39	− 1.46, 0.68	− 0.22	− 1.31, 0.87
2	− 1.18	− 2.22, − 0.14	− 1.52	− 2.62, − 0.42	− 1.07	− 2.12, − 0.02	− 0.81	− 1.93, 0.30
3 +	− 0.34	− 2.25, 1.57	− 0.86	− 2.81, 1.10	− 0.84	− 3.21, 1.54	− 0.51	− 2.87, 1.85
Age of youngest child	Joint *p* = 0.092		Joint *p* = 0.028		Joint *p* = 0.239		Joint *p* = 0.372	
(ref. No child)	–	–	–	–	–	–	–	–
0–2	− 1.09	− 1.97, − 0.21	− 1.48	− 2.46, − 0.49	− 0.96	− 2.09, 0.18	− 0.83	− 2.08, 0.42
3–4	− 0.78	− 2.15, 0.58	− 1.16	− 2.52, 0.20	− 1.01	− 2.36, 0.33	− 0.73	− 1.99, 0.54
5 +	− 0.14	− 1.60, 1.32	− 0.55	− 2.08, 0.99	− 0.07	− 1.37, 1.23	0.28	− 1.03, 1.59

Next Steps cohort COVID-19 survey waves 2–3, ages 30–31. England, September 2020 to March 2021

Model 1: adjusted for ethnicity, housing tenure at ages 13–14, and educational attainment, social class and GHQ score at ages 25–26

Model 2: also adjusted for financial situation before outbreak, and relationship status, living arrangements and employment status at the current wave

Coefficients are linear regression betas, representing the differences in GHQ score between categories. A higher GHQ score represents a higher level of distress

Joint *p* values are adjusted Wald-type tests for all three coefficients

All analyses were weighted and adjusted for the survey design and non-response

#### Main differences

In September–October 2020, GHQ scores did not vary by parent variables in men, but did so in women. Among men, there was evidence that young fathers with older children (aged 5 or more) reported less distress compared with non-fathers in the partially-adjusted model (*b* = −2.48, 95%CI −4.79, −0.18), but this difference was no longer significant in the fully-adjusted model including other circumstances during the pandemic (*b* = −2.12; 95%CI −4.33, 0.09). Among women, compared to non-mothers, living with one child (*b* = −1.17; 95%CI −2.13, −0.20) or two children (*b* = −1.52; 95%CI −2.62, −0.42) was associated with a lower GHQ score in the fully-adjusted model. Living with a child aged 2 or less (*b* = −1.48; 95%CI −2.46, −0.49) was also associated with a lower GHQ score in the fully-adjusted model.

In February–March 2021, there was not strong evidence that GHQ scores varied by parent variables in men or women. Among men, fathers with 3 + children reported more distress compared with non-fathers in the fully adjusted model (*b* = 2.64; 95%CI 0.21, 5.07), but the test for the joint significance of coefficients was not significant (*p* = 0.200). Among women, neither the age (joint *p* = 0.553) or number (joint *p* = 0.372) of children were associated with the GHQ outcome.

#### Interactions

**Fig. 1:**
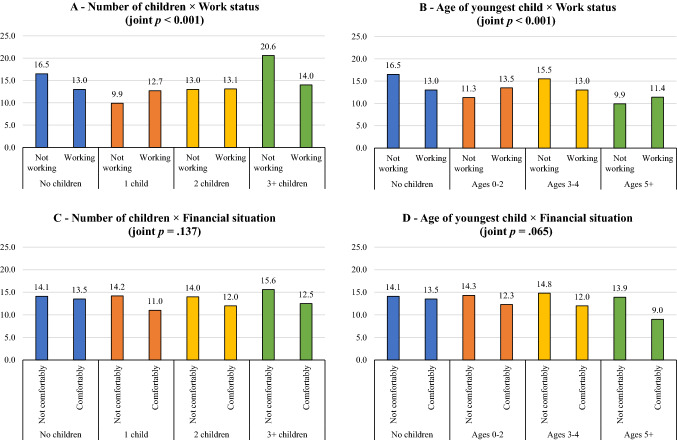
Predicted GHQ scores in Millennial fathers, by work status and financial situation before the outbreak

In men, we found significant interactions by work status and financial situation before the outbreak. In September–October 2020, compared to non-workers, fathers with one child (*b* interaction = 6.19, *p* = 0.001) and fathers with children aged 2 or less (*b* interaction = 5.67, *p* < 0.001) or aged 5 or more (*b* interaction = 4.95, *p* = 0.046) reported more distress if they were working (joint *p*-values < 0.001) (see predicted GHQ scores across groups in Fig. 1).

There was partial evidence that, compared to men living comfortably, fathers with 3 + children (*b* interaction = 2.60, *p* = 0.038, joint *p* = *0.1*37) and fathers with children aged 5 + (*b* interaction = 4.38, *p* = 0.011, joint *p* = 0.065) reported more distress if they were living less than comfortably. In February–March 2021, there was also partial evidence that, compared to men living comfortably, fathers with one child reported more distress if they were living less than comfortably (*b* interaction = 3.03, *p* = 0.022, joint *p* = 0.148).

In women, we found no significant interactions in differences between parent groups by work status, financial situation before the outbreak, or relationship status.

### Sensitivity analysis

Supplementary Table 4 presents the results of the sensitivity analysis examining the role of parent variables in changes in GHQ between September–October 2020 and February–March 2021. Changes in GHQ by February–March 2021 did not vary by parent variables in men, as in the main analyses. Among women, in line with the cross-sectional analyses in September–October 2020, living with one child (*b* = −0.77; 95%CI −1.38, −0.17) or a child aged 2 or less (*b* = −0.88; 95%CI −1.46, −0.30) was associated with a slightly lowered GHQ in February–March 2021 compared with non-mothers.

## Discussion

This study investigated the distribution of psychological distress among Millennial non-parents and parents in September–October 2020 and February–March 2021, matching the start and end periods of the second COVID-19 infection wave in England. Overall, we did not find strong evidence of higher distress among parents at this age across time points, except for young fathers with multiple children in February–March 2021. However, we also found that fatherhood experiences at this age differed by work status, and led some to report higher levels of distress if they were also working. Overall, finding a lack of a “mental health disadvantage” among young parents contrasts with the evidence generated in the general population at the start of the pandemic, which highlighted parents to have had elevated levels of distress in April–May 2020 [29]. Supporting the idea that parents may have quickly recovered after the first wave, another study estimating time trends in anxiety and depressive symptoms in England found that parents recovered more rapidly over time, potentially because children were found to be at low risk of COVID-related health issues [40].

The first of the two differences found across parent groups was that women reported less distress if they were mothers of young children in September–October 2020. This is consistent with trajectories of wellbeing previously observed during motherhood in England, i.e. happiness generally increases before and around childbirth and then decreases to pre-parenthood levels after two years [12]. It may be that the benefits resulting from childbirth outweighed the unique pressures of childrearing during the pandemic. Supporting this, another Israeli study found that younger mothers with greater anxiety over the infant's health perceived greater warmth from infants in April 2020 [41].

The second difference was that young fathers living with 3 + children reported more distress compared with other men in February–March 2021. Interestingly, fathers with children aged 5 + reported less distress compared with other men in September–October 2020, suggesting that it may be dealing with multiple children that was particularly distressing (i.e. not having children at an earlier age). At least two aspects of raising multiple children are likely to negatively impact mental health at this life stage. First, this results in increased levels of economic strain, leaving families at higher risk of not being able to afford material needs [42]. Second, this involves increased caretaking demands, which may put too much stress on the physical and psychological resources of parents [43, 44].

Beyond average differences, we found that compared with non-fathers, fathers with one child or children aged 2 or less were more distressed if they also worked. Many working parents have had to deal with reduced hours, remote work, or new caring responsibilities due to school closures, which has required them to re-define their work and family life balance [23, 45]. A UK study also found that increases in distress among working parents aged 18 + in April–May 2020 were worse among those who applied for unemployment benefits and had difficulties paying their bills, and that these effects were even worse so if they also lived in low-income households [26]. These parents may also have been more likely to experience a lack of affordable food sources, unsafe neighbourhoods with under-resourced schools, and difficulties in obtaining high-quality childcare during the pandemic [46]. Contrasting with studies highlighting mothers to be more vulnerable to financial insecurity at the start of the pandemic, the fact that fathers were more vulnerable here may suggest that the pressures of the “breadwinner” role may be especially strong among new, young fathers compared to older age groups [16, 27].

### Strengths and limitations

This study builds on Next Steps’ large representative sample of Millennials who grew up in England and data on social background and mental health before the pandemic to present robust estimates in young adult parents over the first year of the pandemic. We highlight three limitations. First, the Next Steps COVID-19 survey waves had a low response rate. Despite using weights, there remains a risk that parents who were more affected by the pandemic were under-represented. Samples in September–October 2020 and February–March 2021 may have also varied on other characteristics beyond ethnicity not included in multivariable models, limiting the comparison of findings across time points. The smaller samples finally resulted in a lower capacity to interpret differences as statistically significant, particularly with interactions. Second, the analyses were cross-sectional and we cannot dismiss reverse causation, i.e. that mental health problems led some to avoid parenthood. Third, social desirability and recall biases may have led some to mis-report psychological distress and financial insecurity since the start of the pandemic.

## Conclusion

This study investigated through the prism of parenthood the progression of mental health inequalities in English Millennials during the second COVID-19 wave. The findings support the argument that evidence from the general population at the start of the pandemic may not readily apply to the rapidly changing realities of young parents over time. Whereas parenthood at this age was not a key risk factor of psychological distress, with only the fathers of multiple children reporting more distress compared to non-fathers in 2021, the pandemic has led many young fathers to be disproportionally distressed as a result of new financial insecurity. To support families recovering from the pandemic, the UK government already updated their strategy in 2021 to prioritise re-engaging pupils in school, supporting parents towards employment, and helping families access mental health support [47]. Our findings support the argument that young working parents should be considered a priority for policy. Future rounds of data collection are needed to examine the long-term trajectories of mental health among Millennial parents gleaned from our findings. Other studies are needed to corroborate which father subgroups may be more likely to report elevated distress in this age group, and explore potential age differences in the gendered relationship between parenthood and psychological distress over the life-course.

## Supplementary Information

Below is the link to the electronic supplementary material.

Supplementary file1 (DOCX 65 KB)

## Data Availability

The data used in these analyses are part of the data repository for the Next Steps cohort. Their use for analysis is contingent on formal approval procedures. For more information, please visit https://cls.ucl.ac.uk/cls-studies/next-steps/.
