# Supplementary material for: Parenthood and psychological distress among English Millennials during the second wave of the COVID-19 pandemic: evidence from the Next Steps cohort study
Source: Soc Psychiatry Psychiatr Epidemiol. 2022 Nov 27;58(3):421–30. doi: 10.1007/s00127-022-02392-x (PMC9702632; doi:10.1007/s00127-022-02392-x)
Supplement: Supplementary file 1 — Supplementary file1 (DOCX 65 KB) [file 127_2022_2392_MOESM1_ESM.docx]

**SUPPLEMENTARY MATERIAL**

**Title**

Parenthood and psychological distress among English Millennials during the second wave of the COVID-19 pandemic: evidence from the Next Steps cohort study

**Table of contents**

1. **General Health Questionnaire (GHQ-12) items**
2. **Distribution of covariates across parenthood variables, Wave 3**
   1. In women
   2. In men
3. **Summary of results for interactions**
   1. Work status
   2. Financial situation before the outbreak
   3. Relationship status
4. **Sensitivity Analysis**

**SUPPLEMENTARY TABLE 1.**

**General Health Questionnaire (GHQ-12) items**

| **Have you recently** | Score | | | |
| --- | --- | --- | --- | --- |
|  | 0 | 1 | 2 | 3 |
| 1. Been able to concentrate on what you’re doing? | Better than usual | Same as usual | Less than usual | Much less than usual |
| 2. Lost much sleep over worry? | Not at all | No more than usual | Rather more than usual | Much more than usual |
| 3. Felt you were playing a useful part in things? | More so than usual | Same as usual | Less so than usual | Much less useful |
| 4. Felt capable of making decisions about things? | More so than usual | Same as usual | Less so than usual | Much less capable |
| 5. Felt constantly under strain? | Not at all | No more than usual | Rather more than usual | Much more than usual |
| 6. Felt you couldn’t overcome your difficulties? | Not at all | No more than usual | Rather more than usual | Much more than usual |
| 7. Been able to enjoy your normal day-to-day activities? | More so than usual | Same as usual | Less so than usual | Much less than usual |
| 8. Been able to face up to your problems? | More so than usual | Same as usual | Less so than usual | Much less able |
| 9. Been feeling unhappy and depressed? | Not at all | No more than usual | Rather more than usual | Much more than usual |
| 10. Been losing confidence in yourself? | Not at all | No more than usual | Rather more than usual | Much more than usual |
| 11. Been thinking of yourself as a worthless person? | Not at all | No more than usual | Rather more than usual | Much more than usual |
| 12. Been feeling reasonably happy, all things considered | More so than usual | Same as usual | Less so than usual | Much less than usual |

**SUPPLEMENTARY TABLE 2.1**

**Distribution of covariates across parenthood variables in women. Next Steps cohort COVID-19 survey wave 3, ages 30-31. England, February-March 2021.**

|  |  | **Number of children** | | | **Age of youngest child** | | |
| --- | --- | --- | --- | --- | --- | --- | --- |
|  | **0 children** | **1 child** | **2 children** | **3+ children** | **Age 0-2** | **Age 3-4** | **Age 5+** |
| **Variable** | **N = 1,048** | **N = 411** | **N = 296** | **N = 90** | **N = 398** | **N = 181** | **N = 218** |
|  | **% weighted** | **% weighted** | **% weighted** | **% weighted** | **% weighted** | **% weighted** | **% weighted** |
|  |  |  |  |  |  |  |  |
| **GHQ-12 score in Wave 3, mean** | 15.64 | 15.48 | 14.62 | 15.62 | 15.01 | 14.64 | 15.69 |
|  |  |  |  |  |  |  |  |
| **Ethnicity** |  |  |  |  |  |  |  |
| White | 65.23 | 76.35 | 62.78 | 80.36 | 69.14 | 67.54 | 76.82 |
| Non-White | 34.77 | 23.65 | 37.22 | 19.64 | 30.86 | 32.46 | 23.18 |
|  |  |  |  |  |  |  |  |
| **Homeownership at ages 13-14** |  |  |  |  |  |  |  |
| Yes | 79.51 | 68.96 | 61.97 | 59.93 | 68.42 | 69.81 | 54.78 |
| No | 20.49 | 31.04 | 38.03 | 40.07 | 31.58 | 30.19 | 45.22 |
|  |  |  |  |  |  |  |  |
| **Social class at ages 25-26** |  |  |  |  |  |  |  |
| Never worked, unemployed, or other | 11.83 | 13.99 | 31.70 | 66.03 | 22.04 | 32.63 | 37.39 |
| I: Managerial & professional | 47.79 | 44.78 | 29.96 | 8.60 | 44.11 | 28.29 | 20.11 |
| II: Intermediate & small employers | 20.52 | 19.39 | 19.88 | 15.09 | 19.79 | 23.03 | 14.56 |
| III: Technical & (semi-)routine | 19.86 | 21.84 | 18.46 | 10.28 | 14.06 | 16.05 | 27.94 |
|  |  |  |  |  |  |  |  |
| **Education at ages 25-26** |  |  |  |  |  |  |  |
| Secondary education or less | 27.35 | 32.20 | 48.83 | 65.06 | 37.12 | 49.38 | 50.82 |
| Post-secondary education below degree | 19.32 | 24.50 | 13.84 | 16.31 | 19.09 | 19.89 | 17.22 |
| Degree or above | 53.33 | 43.30 | 37.33 | 18.63 | 43.79 | 30.73 | 31.96 |
|  |  |  |  |  |  |  |  |
| **GHQ-12 at ages 25-26, mean** | 12.16 | 12.28 | 12.08 | 14.25 | 12.85 | 12.29 | 12.07 |
|  |  |  |  |  |  |  |  |
| **Work status in Wave 3** |  |  |  |  |  |  |  |
| Working | 80.68 | 58.87 | 44.20 | 28.33 | 45.74 | 48.88 | 51.20 |
| Not working | 19.32 | 41.13 | 55.80 | 71.67 | 54.26 | 51.12 | 48.80 |
|  |  |  |  |  |  |  |  |
| **Living arrangements in Wave 3** |  |  |  |  |  |  |  |
| Living alone | 11.36 | 21.20 | 27.31 | 24.05 | 12.54 | 30.34 | 37.49 |
| Living with other adults | 88.64 | 78.80 | 72.69 | 75.95 | 87.46 | 69.66 | 62.51 |
|  |  |  |  |  |  |  |  |
| **Relationship status in Wave 3** |  |  |  |  |  |  |  |
| No partner | 21.96 | 19.83 | 21.04 | 13.39 | 10.32 | 25.27 | 28.84 |
| With a partner | 78.04 | 80.17 | 78.96 | 86.61 | 89.68 | 74.73 | 71.16 |
|  |  |  |  |  |  |  |  |
| **Fin. sit. before outbreak in Wave 3** |  |  |  |  |  |  |  |
| Living comfortably | 44.76 | 36.61 | 28.95 | 16.41 | 38.54 | 23.74 | 23.03 |
| Living less than comfortably | 55.24 | 63.39 | 71.05 | 83.59 | 61.46 | 76.26 | 76.97 |
|  |  |  |  |  |  |  |  |

Estimates were produced in the wave-specific complete-case samples.

**SUPPLEMENTARY TABLE 2.2**

**Distribution of covariates across parenthood variables in men. Next Steps cohort COVID-19 survey wave 3, ages 30-31. England, February-March 2021.**

|  |  | **Number of children** | | | **Age of youngest child** | | |
| --- | --- | --- | --- | --- | --- | --- | --- |
|  | **0 children** | **1 child** | **2 children** | **3+ children** | **Age 0-2** | **Age 3-4** | **Age 5+** |
| **Variable** | **N = 790** | **N = 138** | **N = 100** | **N = 26** | **N = 159** | **N = 57** | **N = 48** |
|  | **% weighted** | **% weighted** | **% weighted** | **% weighted** | **% weighted** | **% weighted** | **% weighted** |
|  |  |  |  |  |  |  |  |
| **GHQ-12 score in Wave 3, mean** | 13.98 | 13.68 | 12.72 | 15.77 | 13.72 | 12.71 | 14.66 |
|  |  |  |  |  |  |  |  |
| **Ethnicity** |  |  |  |  |  |  |  |
| White | 73.31 | 60.07 | 92.59 | 47.27 | 57.97 | 86.72 | 82.59 |
| Non-White | 26.69 | 39.93 | 7.41 | 52.73 | 42.03 | 13.28 | 17.41 |
|  |  |  |  |  |  |  |  |
| **Homeownership at ages 13-14** |  |  |  |  |  |  |  |
| Yes | 83.07 | 71.98 | 68.22 | 78.34 | 74.71 | 80.89 | 54.05 |
| No | 16.93 | 28.02 | 31.78 | 21.66 | 25.29 | 19.11 | 45.95 |
|  |  |  |  |  |  |  |  |
| **Social class at ages 25-26** |  |  |  |  |  |  |  |
| Never worked, unemployed, or other | 17.54 | 12.96 | 1.53 | 44.51 | 17.16 | 5.41 | 14.53 |
| I: Managerial & professional | 43.49 | 44.40 | 52.39 | 16.26 | 41.34 | 50.49 | 37.95 |
| II: Intermediate & small employers | 17.11 | 12.79 | 23.76 | 9.45 | 16.14 | 11.53 | 18.21 |
| III: Technical & (semi-)routine | 21.86 | 29.85 | 22.32 | 29.78 | 25.36 | 32.57 | 29.31 |
|  |  |  |  |  |  |  |  |
| **Education at ages 25-26** |  |  |  |  |  |  |  |
| Secondary education or less | 35.88 | 37.34 | 51.39 | 42.52 | 38.56 | 47.40 | 50.48 |
| Post-secondary education below degree | 20.02 | 16.92 | 10.64 | 2.77 | 7.76 | 21.56 | 19.78 |
| Degree or above | 44.10 | 45.74 | 37.97 | 54.71 | 53.68 | 31.04 | 29.74 |
|  |  |  |  |  |  |  |  |
| **GHQ-12 at ages 25-26, mean** | 11.80 | 10.24 | 8.37 | 9.38 | 10.05 | 8.98 | 8.35 |
|  |  |  |  |  |  |  |  |
| **Work status in Wave 3** |  |  |  |  |  |  |  |
| Working | 81.60 | 84.99 | 93.11 | 82.48 | 91.40 | 85.18 | 75.50 |
| Not working | 18.40 | 15.01 | 6.89 | 17.52 | 8.60 | 14.82 | 24.50 |
|  |  |  |  |  |  |  |  |
| **Fin. sit. before outbreak in Wave 3** |  |  |  |  |  |  |  |
| Living comfortably | 42.46 | 30.73 | 23.61 | 54.81 | 37.17 | 19.28 | 29.73 |
| Living less than comfortably | 57.54 | 69.27 | 76.39 | 45.19 | 62.83 | 80.72 | 70.27 |
|  |  |  |  |  |  |  |  |

Estimates were produced in the wave-specific complete-case samples.

**SUPPLEMENTARY TABLE 3.1**

**Summary of interaction tests with work status (employed or self-employed *versus* not).**

|  | **Wave 2**  **Sep.-Oct.2020** | | **Wave 3**  **Feb.-Mar. 2021** | |
| --- | --- | --- | --- | --- |
|  | **Men** | **Women** | **Men** | **Women** |
|  | **N = 994** | **N = 1,824** | **N = 1,054** | **N = 1,845** |
|  | **p-value** | **p-value** | **p-value** | **p-value** |
|  |  |  |  |  |
| **Number of children** | *Joint p < 0.001* | *Joint p = 0.651* | *Joint p = 0.920* | *Joint p = 0.544* |
|  |  |  |  |  |
| 1 child # working | 0.001 | 0.806 | 0.526 | 0.590 |
| 2 children # working | 0.075 | 0.841 | 0.602 | 0.289 |
| 3+ children # working | 0.091 | 0.203 | 0.876 | 0.463 |
|  |  |  |  |  |
| **Age of youngest child** | *Joint p < 0.001* | *Joint p = 0.784* | *Joint p = 0.772* | *Joint p = 0.810* |
|  |  |  |  |  |
| 0-2 years old # working | <0.001 | 0.434 | 0.324 | 0.813 |
| 3-4 years old # working | 0.711 | 0.713 | 0.935 | 0.344 |
| 5+ years old # working | 0.046 | 0.738 | 0.822 | 0.641 |
|  |  |  |  |  |

Interactions were tested based on the fully-adjusted models presented in Tables 2 and 3.

**SUPPLEMENTARY TABLE 3.2**

**Summary of interaction tests with financial situation before the outbreak (living comfortably *versus* not).**

|  | **Wave 2**  **Sep.-Oct.2020** | | **Wave 3**  **Feb.-Mar. 2021** | |
| --- | --- | --- | --- | --- |
|  | **Men** | **Women** | **Men** | **Women** |
|  | **N = 994** | **N = 1,824** | **N = 1,054** | **N = 1,845** |
|  | **p-value** | **p-value** | **p-value** | **p-value** |
|  |  |  |  |  |
| **Number of children** | *Joint p = 0.137* | *Joint p = 0.600* | *Joint p = 0.148* | *Joint p = 0.284* |
|  |  |  |  |  |
| 1 child # living less than comfortably | 0.067 | 0.785 | 0.022 | 0.964 |
| 2 children # living less than comfortably | 0.323 | 0.179 | 0.346 | 0.148 |
| 3+ children # living less than comfortably | 0.038 | 0.866 | 0.997 | 0.158 |
|  |  |  |  |  |
| **Age of youngest child** | *Joint p = 0.065* | *Joint p = 0.802* | *Joint p = 0.436* | *Joint p = 0.444* |
|  |  |  |  |  |
| 0-2 years old # living less than comfortably | 0.234 | 0.423 | 0.110 | 0.155 |
| 3-4 years old # living less than comfortably | 0.181 | 0.618 | 0.390 | 0.633 |
| 5+ years old # living less than comfortably | 0.011 | 0.823 | 0.872 | 0.896 |
|  |  |  |  |  |

Interactions were tested based on the fully-adjusted models presented in Tables 2 and 3.

**SUPPLEMENTARY TABLE 3.3**

**Summary of interaction tests with relationship status (in couple *versus* not) among women.**

|  | **Wave 2**  **Sep.-Oct.2020** | **Wave 3**  **Feb.-Mar. 2021** |
| --- | --- | --- |
|  | **Women** | **Women** |
|  | **N = 1,824** | **N = 1,845** |
|  | **p-value** | **p-value** |
|  |  |  |
| **Number of children** | *Joint p = 0.793* | *Joint p = 0.184* |
|  |  |  |
| 1 child # in couple | 0.970 | 0.571 |
| 2 children # in couple | 0.629 | 0.150 |
| 3+ children # in couple | 0.483 | 0.296 |
|  |  |  |
| **Age of youngest child** | *Joint p = 0.712* | *Joint p= 0.519* |
|  |  |  |
| 0-2 years old # in couple | 0.602 | 0.141 |
| 3-4 years old # in couple | 0.680 | 0.615 |
| 5+ years old # in couple | 0.441 | 0.743 |
|  |  |  |

Interactions were tested based on the fully-adjusted models presented in Tables 2 and 3.

**SUPPLEMENTARY TABLE 4**

**Sensitivity Analysis. Association between parent characteristics at COVID-19 survey wave 2 and psychological distress (GHQ 0-36 score) at wave 3.**

|  | **Men (n=812)** | | | | **Women (n=1540)** | | | |
| --- | --- | --- | --- | --- | --- | --- | --- | --- |
| **Variables** | **Model 1**  **+ partially-adjusted** | | **Model 2**  **+ COVID covariates** | | **Model 1**  **+ partially-adjusted** | | **Model 2**  **+ COVID covariates** | |
|  | **B** | **95%CI** | **B** | **95%CI** | **B** | **95%CI** | **B** | **95%CI** |
|  |  |  |  |  |  |  |  |  |
| **Number of children at wave 2** | *Joint p =0.568* | | *Joint p =0.486* | | *Joint p =* *0.121* | | *Joint p = 0.081* | |
| (ref. No child) | --- | --- | --- | --- | --- | --- | --- | --- |
| 1 | -0.39 | -1.34, 0.56 | -0.15 | -1.13, 0.82 | -0.68 | -1.27, -0.09 | -0.77 | -1.38, -0.17 |
| 2 | 0.42 | -0.70, 1.53 | 0.68 | -0.46, 1.81 | -0.15 | -0.86, 0.55 | -0.26 | -0.97, 0.44 |
| 3+ | 0.83 | -1.21, 2.88 | 1.06 | -1.01, 3.13 | 0.46 | -1.12, 2.04 | 0.30 | -1.28, 1.87 |
|  |  |  |  |  |  |  |  |  |
|  |  |  |  |  |  |  |  |  |
| **Age of youngest child at wave 2** | *Joint p =0.459* | | *Joint p =0.403* | | *Joint p =* *0.058* | | *Joint p =0.004* | |
| (ref. No child) | --- | --- | --- | --- | --- | --- | --- | --- |
| 0-2 | 0.18 | -0.69, 1.04 | 0.44 | -0.45, 1.34 | -0.66 | -1.23, -0.09 | -0.88 | -1.46, -0.30 |
| 3-4 | -0.88 | -2.29, 0.53 | -0.64 | -2.07, 0.80 | -0.29 | -1.25, 0.67 | -0.45 | -1.42, 0.53 |
| 5+ | 0.57 | -1.08, 2.23 | 0.70 | -0.98, 2.38 | 0.52 | -0.45, 1.48 | 0.70 | -0.26, 1.66 |
|  |  |  |  |  |  |  |  |  |

Model 1: adjusted for ethnicity, housing tenure at ages 13-14, and educational attainment, social class, GHQ score at ages 25-26 and GHQ score at COVID-19 survey wave 2.

Model 2: also adjusted for financial situation before outbreak, and employment status at COVID-19 survey wave 3.

Coefficients are linear regression betas, representing the differences in GHQ score between categories. A higher GHQ score represents a higher level of distress.

Joint *p* values are adjusted Wald-type tests for all three coefficients.

All analyses were weighted and adjusted for the survey design and non-response.
